# Monoclonal antibodies specific for the hemagglutinin-neuraminidase protein define neutralizing epitopes specific for Newcastle disease virus genotype 2.VII from Egypt

**DOI:** 10.1186/s12985-021-01540-0

**Published:** 2021-04-26

**Authors:** Ibrahim Moharam, Olayinka Asala, Sven Reiche, Hafez Hafez, Martin Beer, Timm Harder, Christian Grund

**Affiliations:** 1 Institute for Diagnostic Virology, Friedrich-Loeffler-Institute, Südufer 10, 17493 Greifswald-Insel Riems, Germany; 2 Department of Birds and Rabbits Medicine, University of Sadat City, Monufia, Egypt; 3 Viral Vaccines Production Division, National Veterinary Research Institute, Vom, Nigeria; 4 Department of Experimental Animal Facilities and Biorisk Management, Friedrich-Loeffler-Institute, Greifswald, Germany; 5 Institute of Poultry Disease, Freie Universität Berlin, Berlin, Germany

**Keywords:** Monoclonal antibody, Newcastle disease virus, Genotype 2.VII, Antigenicity, Hemagglutinin-Neuraminidase protein, Conformational epitopes

## Abstract

**Background:**

Newcastle disease is a devastating disease in poultry caused by virulent Newcastle disease virus (NDV), a paramyxovirus endemic in many regions of the world despite intensive vaccination. Phylogenetic analyses reveal ongoing evolution of the predominant circulating genotype 2.VII, and the relevance of potential antigenic drift is under discussion. To investigate variation within neutralization-sensitive epitopes within the protein responsible for receptor binding, i.e. the Hemagglutinin-Neuraminidase (HN) spike protein, we were interested in establishing genotype-specific monoclonal antibodies (MAbs).

**Methods:**

An HN-enriched fraction of a gradient-purified NDV genotype 2.VII was prepared and successfully employed to induce antibodies in BalbC mice that recognize conformationally intact sites reactive by haemagglutination inhibition (HI). For subsequent screening of mouse hybridoma cultures, an NDV-ELISA was established that utilizes Concanavalin A (ConA-ELISA) coupled glycoproteins proven to present conformation-dependent epitopes.

**Results:**

Six out of nine selected MAbs were able to block receptor binding as demonstrated by HI activity. One MAb recognized an epitope only present in the homologue virus, while four other MAbs showed weak reactivity to selected other genotypes. On the other hand, one broadly cross-reacting MAb reacted with all genotypes tested and resembled the reactivity profile of genotype-specific polyclonal antibody preparations that point to minor antigenic differences between tested NDV genotpyes.

**Conclusions:**

These results point to the concurrent presence of variable and conserved epitopes within the HN molecule of NDV. The described protocol should help to generate MAbs against a variety of NDV strains and to enable in depth analysis of the antigenic profiles of different genotypes.

**Supplementary Information:**

The online version contains supplementary material available at 10.1186/s12985-021-01540-0.

## Introduction

Newcastle disease virus (syn. avian orthoavulavirus-1; avian paramyxovirus-1 (APMV-1), NDV) is a member of the family of Paramyxoviridae within the genus Orthoavulavirus, which together with the genera Meta- and Paraavulavirus form the subfamily Avulavirinae [1]. Like other members of the order of Mononegavirales, NDV is an enveloped virus with an RNA genome of negative polarity. The genome size of either 15,186, 15,192 or 15,198 nucleotides [2] encodes for six structural proteins [3, 4] with two outer spike glycoproteins, the hemagglutinin-neuraminidase- (HN) and fusion protein (F), that facilitate attachment to and subsequent entry into the host cell. The latter protein is translated as a precursor molecule (F0) and cleaved by cellular proteases into disulfide bond-linked subunits F1 and F2 [5, 6]. Both spike proteins are immunogenic and induce protective immunity in the host [7–9]. The dominating structural protein of the virion however is the nucleoprotein (NP), which enwraps the viral RNA into a helical capsid and together with the phospho (P)-protein and the large (L) protein forms the viral polymerase complex. The matrix (M) protein is positioned underneath the viral membrane and is considered to stabilize the virion architecture. In addition, two regulatory proteins, V and W, are integrated into the virion [10, 11].

In poultry, virulent NDV strains induce Newcastle disease, a systemic infection with mortality of up to 100% in chickens. Introducing avirulent APMV-1 strains as ND vaccines in the late 1940ies was a hallmark for protecting chickens and turkeys from disease [12], and today ND vaccination is fundamental for protecting poultry worldwide. Nevertheless, the disease is endemic in many regions of the world. Lately, inadequate protection by established but old vaccine strains against currently circulating NDV strains has been proposed [13–15]. This hypothesis is supported by genetic analysis: Circulating NDV strains reveal a tremendous heterogeneity and strains can be divided into two genetic classes (1–2) with one (1.I) and 21 (2.I – 2.XXI) recognized genotypes, respectively [16]. Whereas the established vaccine strains belong to genotype 2.I and 2.II, genotype 2.VII dominates the current panzootic in Asia and the Middle East [17, 18]. Homology between the two genotypes for F and HN spike proteins, responsible for eliciting protective immune response in the host [19], is low: 75–79% and 71–75% on the nucleotide level and 85–89% and 84–88% on the level of amino acids. Despite the genetic variation, antigenically NDV still forms a single homogenous serotype [20, 21]. Based on the haemagglutination inhibition test (HI) that detects HN-specific antibodies that block virus binding to sialic acid receptors on erythrocytes or serum neutralization tests, polyclonal sera can differentiate between serotypes of the subfamily Avulavirinae [22, 23] but cannot distinguish between NDV genotypes [21]. However, by monoclonal antibodies (MAbs) differences in specific epitopes have been recognized [24–26]. Profiling of a battery of MAbs raised against NDV-Ulster 2 C (genotype 2.I.2) and pigeon type paramyxovirus (genotype 2.VI) to a total of 1526 NDV isolates allowed distinction of different groups but also revealed considerable heterogeneity within groups [27]. Further studies with neutralizing MAbs to NDV strain Australia-Victoria (AV) (genotype 2.I.1.1) recognized seven different conformation dependent antigenic sites within the HN protein. Two sites conveyed virus neutralization only (sites 3 and 4), while MAbs binding to other sites inhibited HA activity only (sites 1 and 14) or both HA and NA activity (sites 2, 12 and 23) [28–33]. Studies with MAbs established with the apathogenic strain NDV-D26 (genotype 2.I.1.1) established three different epitopes sensitive for both HI and NI activity of MAbs and mapped these sites to different amino acids when escape variants were analyzed [34]. Likewise HI-positive MAb AVS-I, raised against the avirulent LaSota vaccine strain (genotype 2.II) [35] mapped to aa residue 570 that was close to but not part of the other described epitopes [36]. This indicates that NDV strains might express at least slightly different neutralizing epitope patterns. Information for such epitopes of NDV genotype 2.VII is not available.

In order to characterize antigenic sites of circulating NDV genotype 2.VII, we generated genotype-specific MAbs that are able to block biologically active sites of the HN protein, i.e. antibodies that are able to neutralize infectivity and/or block HA activity. For this approach biophysically enriched HN protein fraction of purified virus proved to be an efficient antigen and the applied Concanavalin A (ConA)-ELISA technique that binds antigen not directly on the plate but coupled by the lectin, provided a high throughput system suitable to detect antibodies to conformation sensitive sites. The resulting MAbs were used to recognize unique neutralizing epitopes of NDV genotype 2.VII.

## Material and methods

### Viruses and sera

NDV strain chicken/EGY/NR730/2016 (NR730; GenBank Acc. no. MH899939) was isolated from an ND vaccinated layer flock in Egypt suffering from respiratory distress. The virus was characterized as velogenic having an intracerebral pathogenicity index (ICPI) of 1.8 and belonged to genotype 2.VII.1.1 (formerly 2.VIIb) [37]. Pigeon type paramyxovirus-1 (pigeon/Germany/R75/1998), genotype 2.VI, was derived from the repository of the ND reference laboratory at the FLI (Acc. No. KJ736742) and NDV/clone 30, genotype 2.II, was derived from a commercial vaccine (MSD, New Jersey, USA). As a source of polyclonal reference antibodies, watery egg yolk preparation from eggs from specific pathogen free (SPF) chickens was used, immunized repeatedly with specified inactivated NDV antigens. A monospecific rabbit α-NDV-HN serum and α-NDV-F serum [38] were used for specific detection of NDV-HN protein by western blot (WB) analysis. Immunizations were carried out in accordance with the legally approved protocol (MV-LALLF- 7221.3–2.5–010/10).

### Virus propagation and purification

Virus was propagated in embryonated SPF chicken eggs (ECE) as described [39] (VALO BioMedia GmbH, Osterholz-Scharmbeck, Lower Saxony, Germany). Amino-allantoic fluid (AAF) was harvested on day 3 post infection (dpi) and purified by sucrose gradient ultra-centrifugation. Briefly, debris was cleared from AAF by low speed centrifugation (30 min at 10,976 × g; 10,000 rpm Rotor JA-10; Beckman Coulter, Brea, California, USA). Then virus was spun down by ultra-centrifugation (1.5 h at 96,281 × g; 28,000 rpm, 32Ti Rotor, Beckman Coulter). Virus pellets from 6 tubes were re-suspended in a total of 45 mL of phosphate buffered saline (PBS, pH 7.2) containing 1 M KCl (KCl-PBS) before adding 15 mL of the virus suspension on top of a discontinuous sucrose gradient (30–60%, in KCl-PBS). Visible bands forming after ultra-centrifugation overnight (96,281×*g*; 28,000 rpm, 32Ti Rotor; Beckman Coulter) were collected and diluted 1:5 in KCl-PBS before pelleting the virus by ultra-centrifugation for 1.5 h (96,281×*g*; 28,000 rpm, 32Ti Rotor; Beckman Coulter). The final virus pellets, representing a total of 228 mL AAF were collected and resuspended in 1.5 ml KCl-PBS. The protein concentration of the obtained virus suspension was determined according to Bradford using the Roti®-Quant protein quantitation assay (Carl Roth GmbH, Karlsruhe, Baden-Württemberg, Germany) following the producer’s instructions. Haemagglutination activity was determined using the HA test according to standard procedures [39]. For antigen preparation of pigeon type paramyxovirus (PPMV-1) R75/98 and vaccine type APMV-1 clone 30 purification was done accordingly, but virus was resuspended in PBS.

### Enrichment of HN protein

Separation of NDV spike proteins was done as described by [40]. Briefly, the protein concentration of the purified virus was adjusted to 1.5 mg/mL in KCl-PBS before 0.1 mL Triton X-100 in PBS (20% (v/v)) was added, gently mixed and kept at room temperature (RT) for 20 min. The suspension was centrifuged (20 min at 10,000×*g*, 9703 rpm, A-4-81-11 Rotor, (Eppendorf, Hamburg, Germany)) and the obtained pellet (p1) was resuspended in 1 mL PBS (0.01 M, pH 7.2) and kept for analysis, whereas the supernatant was further cleared by ultra-centrifugation at high speed (1 h at 200,000×*g*, 55,000 rpm Rotor TSL 55 (Beckman Coulter, Brea, California, USA)) for 1 h. Again the pellet (p2) was kept for analysis after resuspension in 0.2 mL PBS. The supernatant was collected and dialyzed against 0.01 M phosphate buffer in order to remove the potassium chloride from the buffer used during purification. Any precipitate that formed during dialysis was sedimented by centrifugation (20 min at 10,000×*g*, 9703 rpm, A-4-81-11 Rotor (Eppendorf, Hamburg, Germany)) and the pellet was resuspended in 0.1 mL PBS (p3). The supernatant of the dialyzed material (s3) was the fraction that was subsequently used as antigen for immunization.

### SDS-PAGE and western blot

Proteins were separated under denaturing conditions in 10% sodium dodecyl sulphate (SDS) polyacrylamide gels using a minigel system (Biorad, Hercules, California, USA) according to standard guides (http://www.bio-rad.com/webroot/web/pdf/lsr/literature/Bulletin_6040.pdf). Shortly, samples were diluted in sample buffer (Roti load® (Carl Roth GmbH, Karlsruhe, Baden-Württemberg, Germany)) heated at 100 °C for five minutes before adding 16 µL per lane into the gel pocket. Protein separation was conducted applying constant voltage setting (200 V) and the gel was either stained by Coomassie blue (Biorad, Hercules, California, USA) or proteins were blotted on a nitrocellulose membrane (Amersham™ Protran, Cytiva, Marlborough, MA, USA) applying constant voltage setting (15 V) for 1.5 h. For western blot analysis the membrane was blocked for one hour with 1% skimmed milk powder in 0.025% Tween 20 in PBS (PBS-Tween) and subsequently incubated with target specific antibodies over night at 4 °C. After washing three times with PBS-Tween, blots were incubated with peroxidase (POD) labeled species-specific anti-immune globulin G (IgG) or immune globulin Y (IgY) conjugates (Sigma-Aldrich, St. Louis, Missouri, USA) for 1 h at RT. After washing three times, peroxidase activity was visualized by chemiluminescence using SuperSignal West Pico Chemiluminescent Substrate (Thermo Scientific™. Waltham, Massachusetts, USA) and the Chemi Doc XRS + imaging system (Bio-Rad, Hercules, California, USA).

### Mouse inoculation and monoclonal production

Two female BALB/c mice were immunized five times intraperitoneally with 20 µg of purified protein fraction (S3) mixed with an equal amount of GERBU Adjuvant MM (GERBU Biotechnik, Heidelberg, Germany) over a period of 26 weeks, with boost immunizations at weeks 4, 7, 11 after the first administration, and four days before extraction of the spleen. Blood samples were taken from the submandibular vein on days 35, 49, and 84 after first immunization (dpi) and at 183 dpi, at the end of the experiment. Four days after the final boost mice had been euthanized, their spleens were removed under aseptic conditions and splenocytes were harvested into serum-free RPMI-1640 medium (Invitrogen, Carlsbad, California, USA/Thermo Scientific™. Waltham, Massachusetts, USA) by using a cell strainer (BD Biosciences, Franklin Lakes, New Jersey, USA). In the presence of polyethylene glycol 1500 (Roche Applied Science, Penzberg, Germany) the isolated splenocytes were fused with murine myeloma SP2/0 cells following a slightly modified standard protocol [41] by using a cell-to-cell ratio of 1:4. Fused spleen cells were seeded in three different cell densities (30,000, 15,000, and 7,500 spleen cells per well, two plates per density) in 96well flat-bottomed plates (Greiner bio-one, Kremsmünster, Austria) and incubated for 10 days (37 °C, 90% RH, and 5% CO2) by using complete RPMI-1640 culture medium 10% FCS (Fischer Scientific, Hampton, New Hampshire, USA), 1 × MEM non-essential amino acids, 2 mM L-glutamine, 1 mM sodium pyruvate (Invitrogen, Carlsbad, California, USA/Thermo Scientific™. Waltham, Massachusetts, USA) supplemented with 1 × BM Condimed H1 (Hybridoma Cloning Supplement, Sigma-Aldrich). For selection of growing hybridoma clones the complete medium was additionally supplemented with 1 × HAT Media Supplement (50×) Hybri-Max™ (Sigma-Aldrich). Growing cultures were screened for specific antibodies by Con-A ELISA, IF and HI. The haemaglutination inhibition test (HI) was used to determine the haemagglutination inhibition activity according to standard procedures [39]. For generating MAb producing cell clones, cells from positive cultures were cloned at least twice by limiting dilution (0.1 cells per well) in complete RPMI-1640 medium supplemented with 1 × HT Media Supplement (50 ×) Hybri-Max™ (Sigma-Aldrich). Final clones were adapted to complete RPMI-1640 medium without any further supplements.

### Con-A ELISA

The ELISA procedure was done according to a previously published protocol [42, 43]. Briefly, ELISA plates (Immunolon II, Thermo Scientific™. Waltham, Massachusetts, USA) were pretreated with Concanavalin A (ConA) (Carl Roth GmbH) by adding 50 µl of Con-A (50 µg/mL in PBS) to each well. After incubation for 1 h at RT plates were washed three times using PBS-Tween and coated with 50 µL (20 µg/mL) pre-treated antigen. For preparation of the antigen, gradient purified virus stock at a concentration of 200 µg/mL was incubated with TritonX100 (1% v/v) for 45 min at RT and subsequently diluted 1:10 with PBS for coating the plates for 1 h. Thereafter, plates were washed three times with PBS-Tween and blocked with 1% FCS in PBS for 30 min at RT. Test sera (50 µL/well) at indicated dilutions were incubated for 30 min at RT and after three washings with PBS-Tween, plates were incubated with POD labeled species specific anti-IgG or IgY conjugates (50 µL/well) for 30 min at RT. After washing the plates three times with PBS-Tween, bound antibodies were visualized by incubation with o-Phenylenediamine dihydrochloride (Sigma-Aldrich). (50 µL/(100 µL/well, 1 mg/mL in 0.05 M phosphate-citrate buffer) for 20 min. Colour reaction was stopped with 2.5 M H_2_SO_4_. Well density was measured at 492 nm using Sunrise™, Tecan’s microplate readers (Männedor) and the optical f, Zürich, Switzerland).

### Indirect immunofluorescence (IF)

Immunofluorescence test was done with NDV/NR730/16 infected LMH cells (ATCC CRL-2117™) cultivated in 96 well plates (Corning, Corning, New York, USA) grown to 70–80% confluency before infection. Infected plates were fixed with 3.7% formaldehyde after incubation for 24 h and stored at 4 °C for up to four weeks. For use, plates were emptied and treated with TritonX-100 (1% v/v) in PBS (100 µL/well) for 30 min at RT and after flicking off the supernatant, blocked with 1% fetal calf serum in PBS for 30 min at RT. Subsequently, cells were incubated with MAb or indicated sera (50 µL/well) for 1 h at RT. After washing the plates three times with PBS-Tween, wells were incubated with FITC-conjugated anti-species specific IgG or IgY conjugate (Sigma-Aldrich, country) for 1 h at RT. After final washing three times with PBS-Tween, wells were mounted with glycerol in water 1:5 and inspected under the microscope (Eclipse TS100, Nikon, Minato, Tokyo, Japan) with the appropriate filter (excitation 495 nm, emission 525 nm).

### Serum neutralization test (SNT)

Serial dilutions (log_2_) of MAbs, starting with concentrated supernatants were mixed with equal volume of NDV/NR730/16 (100 µL) containing 400 tissue culture infectious dose 50 (TCID_50_). For each dilution row, the last well was kept without serum to serve as virus control. For each test a heat inactivated (56 °C, 30 min) NDV reference serum was included as positive neutralization control. After incubation for 30 min at 37 °C, 50 µL of antibody / virus mixture was transferred in triplicate to 100 µL LMH cells that were cultivated without fetal calf serum but with TPCK treated Trypsin (2 µg/mL) (Sigma-Aldrich) in 96well plates (Corning) at a density 10^6^ cells/cm^2^. The plates were incubated at 37 °C with 5% CO_2_ atmosphere for four days and SNT titer was determined by determining the last dilution without presence of a cytopathic effect (cpe) and calculated according to Reed and Muench [44].

### Haemagglutination inhibition test (HI)

Haemagglutination inhibition test was done according to standard procedures applying four haemagglutinating units, and HI titer are given as last serum dilution (log2) that inhibits agglutination [37]. For analyzing antigenic differences by polyclonal sera, the tests were done in triplicate and repeated in three independent experiments. Results were analyzed by Sigma Plot 11 (Systat Software, INC) applying Kruskal–Wallis One Way Analysis of Variance on Ranks. R values were calculated according to Archetti and Horsefall [45] with R: 0.5, 0.25, 0.13 and 0.06 indicating log2 titer difference of 1, 2, 3 and 4 respectively.

## Results

### HN antigen preparation

**Fig. 1:**
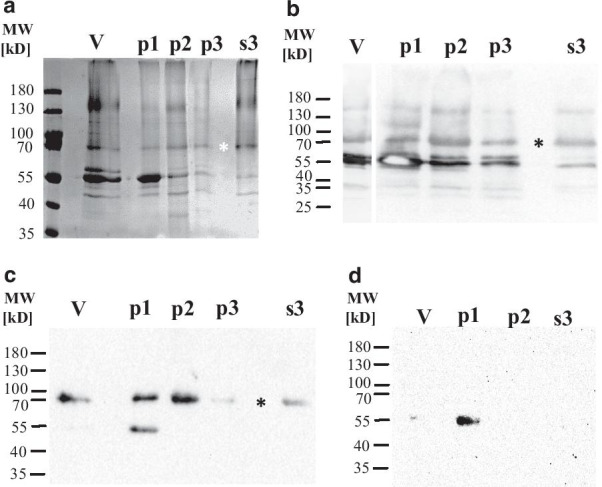
Preparation of HN-enriched fraction from purified NDV/NR730/2016. Purified virus and fractions of the preparation were subjected to SDS-PAGE and subsequent Coomassie staining (**a**) and Western blot analysis using egg yolk preparation of a vaccinated chicken (**b**), HN specific rabbit hyperimmune serum (**c**) or F-specific rabbit hyperimmune serum (**d**). Beside the original gradient purified virus from AAF (V), the discarded pellets after Triton-100 treatment and two successive centrifugation steps (p1 and p2) are shown, together with the insoluble fraction after dialysis (p3) and the final supernatant used for immunisation (s3)

For the antigen preparation, gradient purified virus harvested from AAF (228 ml) was used, that yielded 1.5 mL with an HA activity of 15 (log_2_) and a total protein content of 1.9 mg/mL. Purified virus preparations were dominated in SDS-PAGE and subsequent Coomassie staining by a band at 55 kD, likely representing co-migrating proteins of NP (53 kD), P (53–56 kD) and F1 (55 kD) (Fig. 1a). This protein fraction is recognized as a major immunogenic fraction in the virion also by antibodies of NDV vaccinated chicken (Fig. 1b). In addition, the HN protein is apparent as a ~ 70 kD band by Coomassie staining (Fig. 1a) and in western blotting (Fig. 1b) as indicated by * in the figures. The identity of the HN protein with the observed ~ 70 kD band could be confirmed using a HN-monospecific hyper-immune serum in WB analysis (Fig. 1c). Likewise a minor band at ~ 40 kD representing the M protein, was detected by Coomassie staining (Fig. 1a) and polyclonal chicken antibodies in western blot analysis (Fig. 1b). After disruption of the virus with Triton and subsequent centrifugation (p1), proteins of the 55 kD bands were the dominant fraction of the pellet. Embedded in this band is the F1 protein that was specifically detected by an F-specific hyperimmune serum (Fig. 1d). Subsequent pellets (p2 and p3) and also the final supernatant (S3) still contained proteins of the 55 kD band, detected by Coomassie staining (Fig. 1a) and western blot analysis (Fig. 1b). However, compared to the other proteins in these fractions, the 55 kD band was less prominent. During all centrifugation steps also HN protein was pulled down, but gradually became the dominant fraction in the final supernatant that was used for the immunization (S3) and retained an HA activity of 13 (log_2_). On the other hand, the western blot analysis with the F-specific serum (Fig. 1d) indicated that the F1 protein was no longer present in these later fractions including the final supernatant.

### Establishing the ConA ELISA test system

**Fig. 2:**
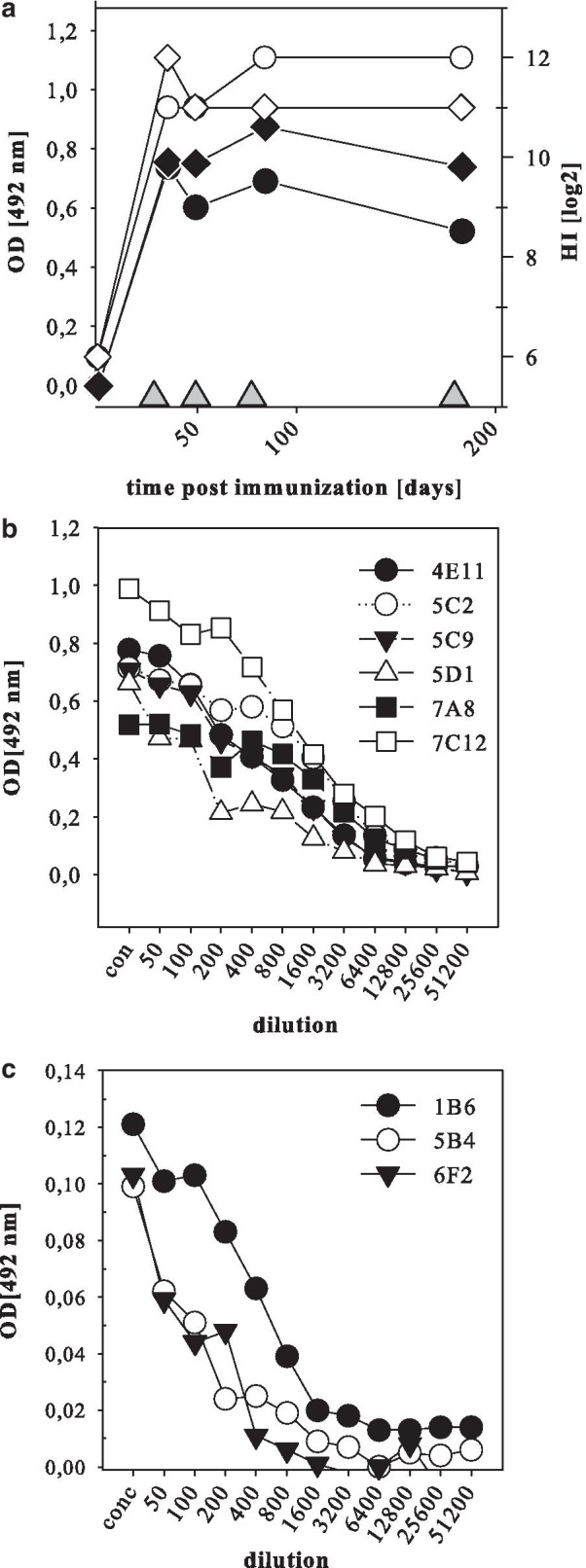
Antibody response of immunized mice and reactivity of obtained MAb. Antibody response of two mice immunized with the S3 antigen preparation were tested at indicated times after the first immunization by HI (filled symbols) and by ConA ELISA (white symbols) (**a**). Time of booster immunizations are given as triangles (filled triangle). In addition, ConA ELISA reactivity of HI positive MAbs (**b**) or HI-negative cloned MAbs (**c**) is given

**Fig. 3:**
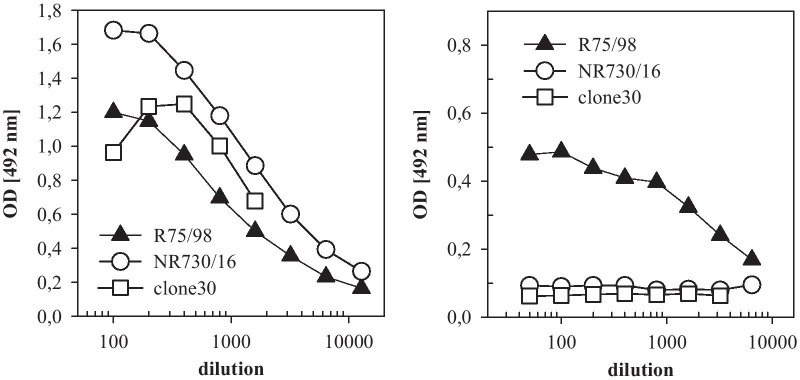
ConA-ELISA presents conformational dependent HN-epitope. Reactivity of MAb 617/161 recognizing specifically genotype 2.VI (PPMV-1) by HI, was tested against different antigens (PPMV-1: R75/98, 2.VII: NR730/16; 2.II: clone 30) by ConA- ELISA (**b**). Coating of plates was verified by homologue polyclonal chicken antibodies (**a**)

Already after the first immunization of two BALB/c mice with the S3 fraction (20 µg/mouse), NDV specific antibodies were detected in blood samples (Fig. 2a). Reactivity by HI (Fig. 2a, filled symbols) indicated that induced antibodies were able to block receptor mediated binding of the virus to erythrocytes, demonstrating that the antigen preparation represents conformationally intact epitopes of the HN protein. Antibody reactivity was also detected by ConA ELISA (Fig. 2a, transparent symbols). However, to verify that the ConA ELISA also detects antibodies to conformation dependent epitopes, the test system was checked with MAb 617/161 [46]. This MAb has HI activity against genotype 2.VI but not genotypes 2.II or 2.VII, and does not react with denatured viral protein in western blot analyses. Hence, it is directed against a conformation-dependent epitope. In ConA-ELISA of plates coated with NDV genotype 2.II (clone 30), genotype 2.VI (pigeon/ DEU/R75/98) or genotype 2.VII chicken/EGY/ NR730/2016), the polyclonal reference antibody preparation was reactive with all three antigens (Fig. 3a). In contrast, but in agreement with the HI data, MAb 617/161 reacted only with genotype 2.VI antigen (Fig. 3b). Having established that the ConA-ELISA is suitable to detect antibodies to conformational epitopes within the HN protein of NDV, this assay was chosen for screening of supernatants from hybridoma cultures.

### Preparation of monoclonal antibodies

For the preparation of hybridoma cells, splenocytes of both mice were harvested four days after the 4th booster immunization, pooled, and half of the cells were used for the fusion. From a total of ten seeded 96well plates, 30 wells yielded supernatants reactive in the Con A-ELISA with an OD reading above 0.2 (0.2–0.7), and virus-specific reactivity could be confirmed by IFT. Supernatants of 6 of the 30 wells also showed reactivity by HI to the homologue NDV NR730 (genotype 2.VII) (HI-titer (log2) between 1 and 5 and were chosen for subcloning and further characterization. In addition, three HI-negative cultures where chosen. Final supernatants from hybridoma cultures obtained after two rounds of cloning by limited dilution were subsequently used for the characterization. The 6 HI-positive hybridoma colonies showed reactivity by ConA-ELISA (Fig. 2b) and retained HI reactivity after single cell cloning (Table 1).

**Table 1:** Reactivity of obtained monoclonal antibodies (MAbs)

MAb	HI (log2)		IF		ConA ELISA [OD 492]		WB		SNT (log2)	Specificity
	NR730	clone 30	NR730	clone 30	NR730	clone 30	NR730	clone 30	NR730	
4E11	6	5	pos	pos	1.79	1.19	neg	n.d	8	HN
5C2	4	neg	pos	neg	1.69	0.17	neg	n.d	neg	HN
5C9	4	neg	pos	neg	1.86	0.08	neg	n.d	7	HN
5D1	3	neg	pos	neg	1.74	0.09	neg	n.d	8	HN
7A8	5	neg	pos	neg	1.55	0.07	neg	n.d	neg	HN
7C12	5	neg	pos	neg	2.04	0.08	neg	n.d	8	HN
1B6	neg	neg	pos	neg	0.18	0.18^#^	55 kD	55 kD	neg	NP/P*
5B4	neg	neg	pos	neg	0.16	0.06^#^	55 kD	neg	neg	NP/P*
6F2	neg	neg	pos	neg	0.16	0.05^#^	55 kD	neg	neg	NP/P*

NR730: virulent Egyptian NDV isolate of genotype 2.VII, directly; clone 30: avirulent NDV vaccine strain of genotype 2.II

(#): directly coated ELISA plates; pos: positive reaction; neg: negative reaction

(*): deduced from western blot analysis due to co-migration of NP and P-protein

### Analysis of cross reactivity reveals unique epitope patterns in the HN of genotype 2.VII

**Fig. 4:**
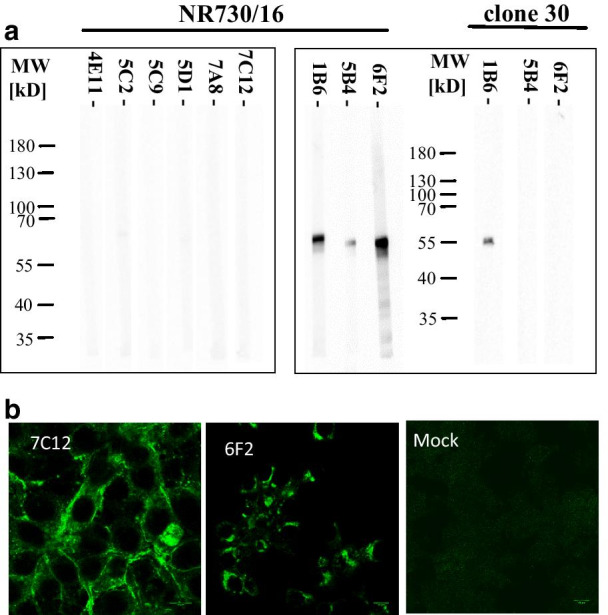
Analysis of reactivity profile of MAbs. All MAbs were tested by WB-analysis (**a**). Boxes are giving results of the HI-reactive MAbs (left) and HI-negative MAbs (right). Beside testing with homologous virus antigen (NR750/16 genotype 2.VII), cross-reactivity of MAb (1B6) to genotype 2.II NDV was confirmed with heterologous clone 30 antigen. Besides reactivity was tested by IFT with the homologous virus NR750/16 (**b**)

Initial analysis of the MAbs addressed the reactivity in different test systems and considered cross-reactivity to NDV vaccine strain clone 30, genotype 2.II. The majority of MAbs reacted specifically with the homologues genotype 2.VII virus (NR730) (Table 1). In addition, one of the HI-positive MAb (4E11) was able to block haemagglutination of clone 30 as well, although at two log_2_ titer steps lower, compared to the homologous NR730 antigen. Reactivity was confirmed by IFT with specific reactivity with NR730 infected cells (Fig. 4b) and cross-reactivity to clone 30 for MAb 4E11, respectively (Table 1). By ConA-ELISA, the HI-positive MAbs showed a strong reactivity to the homologous NR730 genotype VII virus (Fig. 2b) and again only MAb 4E11 cross-reacted with heterologous vaccine strain clone 30 (Table 1). In contrast, ConA-ELISA reactivity of the three HI-negative MAbs was low (Fig. 2c). Interestingly, two MAbs (5B4, 6F2) reacted only with the homologous genotype 2.VII virus (NR730/16), while the third MAb (1B6) also reacted with a genotype 2.II vaccine strain (clone30). This reactivity profile could be observed by ELISA (Table 1) and confirmed by western blot analysis (Fig. 4a): All three HI-negative MAbs reacted with virus proteins migrating at ~ 55 kD of NR730 and, analogously to the ELISA, only MAb 1B6 showed cross-reactivity to clone 30. However, as NP, P and F1 co-migrate on SDS-PAGE gels, it is not possible to draw conclusions with regard to the specificity of the MAbs from our western blot results. In contrast, none of the HI-positive MAbs were reactive by western blot analysis (Fig. 4a). The final test explored whether the MAbs were able to block infection of NR730 in LMH cells. By SNT four out of the six HI-positive MAbs neutralized viral infectivity at titers comparable to the HI test. In contrast, none of the three MAbs reactive with the 55 kD proteins were able to neutralize homologous NDV NR730.

For the six HI-positive MAbs we extended our analysis of cross-reactivity with six additional strains, representing class 1 and 2 viruses (Table 2). It became clear that the epitope recognized by MAb 4E11 was present in all genotypes tested and represents a well conserved site. For the other five HI-positive MAbs, reactivity profiles were highly specific for genotype 2.VII, even though one MAb (5C2) had residual reactivity to viruses from other genotypes. This includes Herts 33/56, a very early NDV strain not assigned to a specific genotype within class 2 viruses and hence termed genotype 2.0. In addition, low level reactivity was observed with the PPMV-1 virus (genotype 2.VI) and a recent virus of genotype 2.XIV from Nigeria. Apparently, also within genotype 2.VII there are antigenic differences: While three MAb (5C2, 5D1 and 7C12) were reactive with a second genotype 2.VII virus tested, reactivity of MAb 5C9 was fourfold lower with the heterologous virus. For MAb 7C12 reactivity to heterologous dropped to 1 log2. In contrast, polyclonal antibody preparations raised against genotypes 2.I, 2.II, 2.VI, 2.VII and 2.XIV were reactive with all antigens tested (Additional file 1: Fig. S1). For the majority of antigens, titer differences of sera were in the range of 2 log_2_ steps. However, sera raised against genotype 2.I, 2.VI and 2.XIV, respectively, had HI titer differences (dHI) of > 2 (log_2_) to individual antigens, a discrimination not present with other sera. This is reflected when analyzing the antigenic relation of the five antigen / serum pairs (genotype 2.I, 2.II, 2.VI, 2.VII and 2.XIV) by calculating the R values according to Archetti and Horsfall [45]. The majority of R values were between 0.9 (genotype 2.I /2.II) and 0.25 (genotype 2.VI / 2.XIV) (Additional file 2: Table S1). The highest R value was 0.15 (genotype 2.I /2.VI), resembling dHI of almost 3.

**Table 2:**
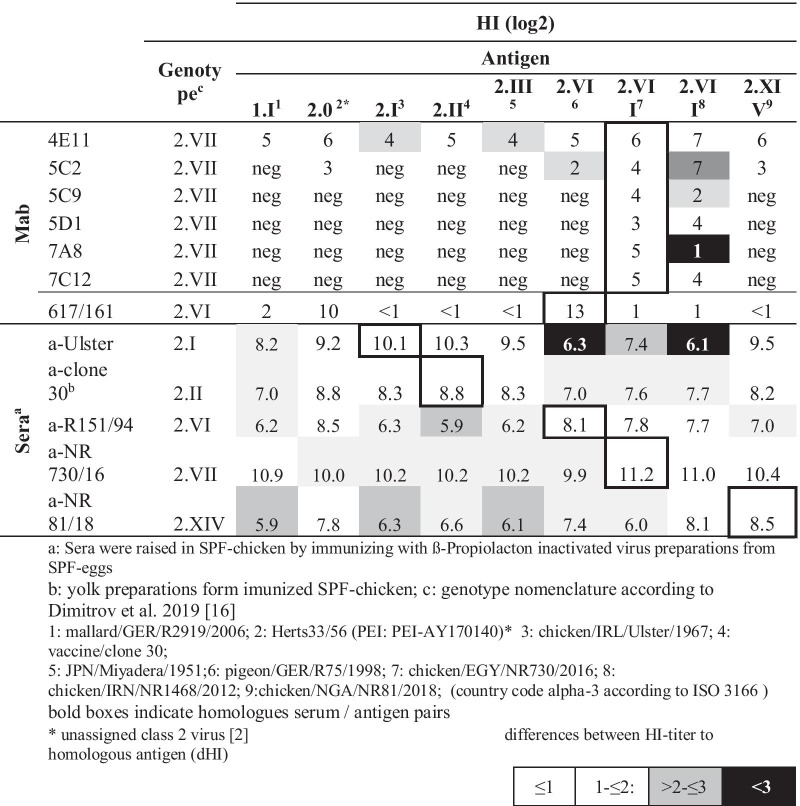
Cross-reactivity by HI assay of HN-specific MAbs compared to polyclonal NDV reference sera

## Discussion

### HN-enriched virus antigen induced antibodies to conformational epitopes

We report on the generation of MAbs to NDV that recognize genotype 2.VII specific epitopes on the HN protein. By enrichment of the HN protein fraction in the antigen preparation used for immunization of mice, we were able to obtain six hybridoma cultures that are reactive by HI, i.e. are able to block the receptor binding site of the HN protein on erythrocytes, and four of them neutralized viral infectivity in LMH cell cultures. In addition, three further selected hybridoma cultures neither showed HI nor neutralizing activity. By western blot analyses, these latter MAbs revealed reactivity against viral proteins that migrate at 55 kD and, hence, do not target HN. In the past, antibodies against NDV have been prepared by using whole, mostly gradient-purified virus preparations [24, 26, 47]. With this approach Lana and colleagues reported that five hybridoma fusion experiments yielded 20 NDV specific MAbs, of which only three were reactive by HI. Earlier reports [35] obtained a single MAb from a total of 184 maintained hybridoma cultures that was specifically reactive with the homologous virus by HI. The described problems to obtain sufficient numbers of MAbs reactive with ND spike proteins might in part be explained by the composition of the viral particle, which is dominated by the highly immunogenic NP protein that fills the entire inner virion [48]. In addition, the M protein, forming an array underneath the viral membrane is abundant in the virion [49]. Both proteins account for ~ 30% of the virus-specific molecular masses in gradient-purified viral preparations when analyzed by nano-LC MALDI-TOF/TOF mass spectrometry, and only about 10% of the virion are HN molecules [11; Karger personal communication]. By disrupting gradient-purified virus by Triton X-100 (2%) and 1 M KCl, Nishikawa and colleagues could increase the fraction of HI reactive HN-specific MAbs, retrieving 9 out of a total of 21 [50]. Our method also used Triton X-100 and KCl disruption, but we subsequently applied ultracentrifugation to enrich HN and deplete M and NP proteins. This procedure was able to pull down a high proportion of the proteins that migrate as a 55 kD band including the NP. The subsequent steps reduced the proportion of the 55 kD proteins further, but were not sufficient to remove all of the proteins: A slight band was still visible by Coomassie-staining (Fig. 1a) and reacted with the polyclonal antibody preparation from a ND vaccinated chicken (Fig. 1b). This might be a result of incomplete separation of the interaction of the HN protein with the NP protein via the matrix protein [51]. Accordingly, three of the selected MAbs were reactive with the 55 kD band by western blot (Fig. 3). However, the western blot with an F-specific serum (Fig. 1d) identified that a large proportion of the F1 protein was in the very first pellet of the antigen preparation, indicating that F protein was not part of the antigen. This would be in line with results of co-immunoprecipitation experiments, showing a direct interaction of F and NP and HN and M [51]. Thus, it is likely that the three HI-negative MAbs are directed against NP or P protein. Further studies on protein specificity will have to address this question and evaluate potential potency in diagnostic approaches. Harsher conditions for particle disruption might have yielded an improved depletion of nucleocapsid proteins but, at the same time, may have denatured HN and destroyed HI epitope conformation.

Overall our aim to produce MAbs capable to block biologically active sites of the HN protein of genotype 2.VII NDV by using an HN-enriched fraction of purified virus proved to be successful. We obtained a sufficient number of HI-positive MAbs already with a single fusion experiment, and two thirds of the selected hybridoma cultures yielded HN-specific MAbs that were able to block receptor binding.

### ConA-ELISA presents biologically intact and relevant antigenic sites of NDV

Due to the complex structure of biologically relevant epitopes of the homo-tetrameric HN protein [52–54], the approach to generate MAbs reactive with biologically activesites depends on native HN protein with the correct conformation, ideally from virus produced in eukaryotic cells ensuring bona fide glycosylation and folding. Reassuring evidence was obtained through the HI-positive antibody response of two mice immunized with enriched HN preparations. However, in order to select the appropriate antibodies, the screening system has to present the test antigen in its native conformation. When using the indirect ELISA techniques, immobilizing antigen directly to the plastic surfaces of the plates likely interferes with the conformational integrity of the antigen, and important epitopes may be unpredictably masked or exposed [55]. To avoid such structural alterations caused by the coating, Russel and colleagues used NDV infected and formalin fixed cells with indirect immunoperoxidase test to visualize specific reactivity [25]. Having the advantage that already staining pattern in this test may give an indication whether MAbs are directed to outer glycoproteins, the test depends on manual inspection of the plates and is more difficult to standardize; in addition, use of formalin may denature sensitive epitopes. To be able to use ELISA technique for high throughput screening, investigators have used antibody- or poly L-lysine-coated plates to capture and thereby preserve the conformation of a viral protein [56, 57]. Our test system relied on the lectin properties of ConA as an anchor for the antigen, a system previously shown to preserve conformational epitopes of viral glycoproteins [42, 43]. By using a genotype 2.VI specific monoclonal antibody that is highly reactive by HI [46], we could demonstrate that the ConA-ELISA preserves NDV epitopes sensitive to receptor blocking. Applying the Con-A-ELISA for screening of the initial hybridoma supernatants and subsequent cloning, we obtained six MAb that were reactive by ELISA and HI, and all six clones could be confirmed by IFT. However, none of the six HI-positive MAb were reactive by WB, which is in line with previous studies that denaturing WB conditions destroyed the conformation of HI sensitive epitopes of the HN molecule. Overall, comparing ELISA reactivity to HI titers, it became apparent that ConA-ELISA was far more sensitive producing titers of well above 1:3200, whereas the highest HI titer was 1:256 (8 log_2_) but without a direct correlation between the two values. It is striking that the MAb with the lowest HI titer (5C2) had comparable or even higher ELISA reactivity than the other MAbs. The ConA-ELISA, in addition, was able to present strain specific epitopes, as five out of six HI-positive MAbs reacted only with NDV genotype 2.VII strain NR730 but not with the vaccine strain clone 30, genotype 2.II, by HI. Thus, using HN-enriched virus preparation in conjunction with ConA-ELISA system that preserves conformational epitopes proved to be efficient to produce specific MAbs that are suitable to identify biologically relevant targets in specific NDV genotypes.

### HN-specific MAbs distinguish strain-specific and broadly cross reacting epitopes

In order to investigate the putative conservation of HI-sensitive epitopes of HN proteins of ND viruses of different genotypes we extended the analysis to cross-reactivity using NDV strains representing distantly related class 1 NDV as well as seven additional class 2 genotype viruses. Only MAb 4E11 was reactive with all 8 different NDV genotypes tested, including the class 1 strain. On the other hand, the majority of HN specific MAbs predominantly reacted with the homologous NDV strain NR730, although within this group of MAbs three distinct reactivity profiles were discernable: (a) MAb 5C2 showed residual cross-reactivity to three other genotypes, (b) three MAbs (5C9, 5D1, 7C12) reacted specifically with two different genotype 2.VII strains, and (c) MAb 7A8 differentiated between the two genotype 2.VII viruses. This points, in summary, to a high degree of flexibility of some HI epitopes, whereas other epitopes seem to be very well preserved. This is corroborated by observations derived from structural analysis of the HN molecule: Crenell and colleagues found that MAbs could interfere with receptor binding, even though the target was not the receptor binding pocket itself [52]. In consequence such sites might not be crucial for the function of the molecule and are allowed to have more structural flexibility. Intrinsic properties of antibodies targeting these sites might then influence the specificity of the reaction, i.e. antibodies with high affinity might be able to bind a broader spectrum of virus strains. On the other hand, epitopes adjacent to or directly at the receptor binding site are part of functionally pivotal structures and have to stay conserved. These epitopes would represent conserved antigenic sites across the genotypes as recognized by the MAb 4E11 which showed HI and neutralization activity. In the viral HN antigen used for immunizing the mice, both types of sites were apparently immunogenic. Considering the number of obtained MAb, i.e. five strain specific versus one MAb specific for a conserved site, the variable sites might have been more immunogenic for mice. In contrast, polyclonal antibody preparations revealed only minor antigenic differences, indicating a balanced presentation of conserved and strain-/genotype-specific antigenic sites for chickens. In consequence polyclonal sera are of limited value when addressing variability of antigenic sites. On the other hand, polyclonal sera raised after immunization of chickens are capable to react to (and neutralize) a broad range of different NDV genotypes; this in turn may also relate to cross-protection against currently circulating field strains after vaccination with genotypically distant strains.

## Conclusion

Further studies on epitope mapping of MAbs will help to elucidate these specific epitopes. It will be interesting to learn whether genotype-specific antibodies recognize known binding sites but have a different amino acid configuration or whether the genotype 2.VII specific epitopes are formed by different sites. Overall our studies highlight the value of MAb to dissect antigenic sites. The established MAbs are the first step to define genotype 2.VII specific neutralizing sites of NDV based on sequence information and should enable phylogenic analysis of antigenic sites in the future, a prerequisite to elucidate whether antigenic drift was the driving factor for evolution of NDV. In addition, well defined MAbs serve as valuable diagnostic tools to specifically detect NDV antigen for example in lateral flow devices or can be used for serological tests. When, for example, applied in blocking ELISA systems genotype specific reagents would allow to differentiate infected from vaccinated individuals (DIVA) by specifically detecting genotype 2.I, 2.II or 2.VII specific antibodies. The presented work flow provides a powerful method to generate and characterize these reagents.

## Supplementary Information

**Additional file 1: Fig. S1**. Antigenic profiling of different NDV genotypes. Sera against five different genotypes were tested by HI against nine different antigens representing eight different NDV genotpyes. Boxplots represent results of three independent tests with three replicates each. Significant differences (p<0.05) to homologues serum, marked by red boxes, are indicated (*).

**Additional file 2: Table S1**. Antigenic relation of NDV genotypes.

## Data Availability

The datasets during and/or analysed during the current study available from the corresponding author on reasonable request.

## Abbreviations

AV: Australia-Victoria
ConA: Concanavalin A
F: Fusion-protein
HI: Haemagglutination inhibition test
HN: Heamagglutinin-Neuraminidase-protein
dHI: HI-titer difference
IF: Indirect immunofluorescence
L: Large-protein
M: Matrix-protein
MAb: Monoclonal antibody
NDV: Newcastle disease virus
NP: Nucleo-protein
OD: Odipical density
POD: Peroxidase
P: Phospho-protein
PPMV-1: Pigeon type paramyxovirus -1
PAGE: Polyacrylamide gel electrophorese
Rpm: Rounds per minute
SNT: Serum neutralization test
SDS: Sodium dodecyl sulphate
SPF-chicken: Specific pathogen free chicken
WB: Western blot
