# Supplementary figures and images for: Monoclonal antibodies specific for the hemagglutinin-neuraminidase protein define neutralizing epitopes specific for Newcastle disease virus genotype 2.VII from Egypt
Source: Virol J. 2021 Apr 26;18:86. doi: 10.1186/s12985-021-01540-0 (PMC8072307; doi:10.1186/s12985-021-01540-0)

## Slide 1
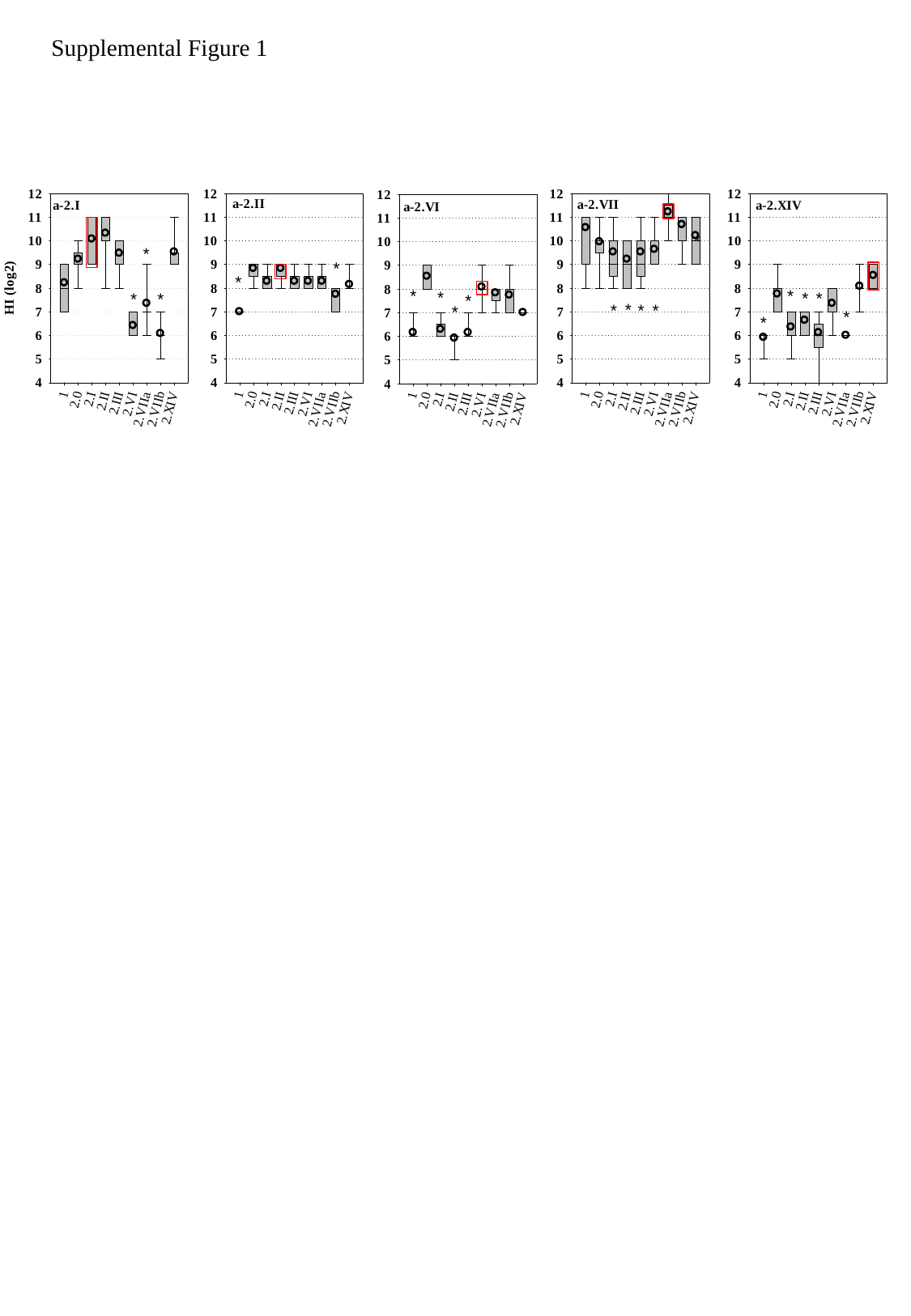

Supplemental Figure 1

Supplement: Supplementary file 1 — Additional file 1: Fig. S1. Antigenic profiling of different NDV genotypes. Sera against five different genotypes were tested by HI against nine different antigens representing eight different NDV genotpyes. Boxplots represent results of three independent tests with three replicates each. Significant differences (p<0.05) to homologues serum, marked by red boxes, are indicated (*). [file 12985_2021_1540_MOESM1_ESM.pptx]
